# Letter from F. K. Bailey, M. D., Joliet

**Published:** 1859-06

**Authors:** 


PROF. BRAINARD:

In the March number of the Journal is an article headed, "Stomatitis Materna—Report of Committee on Practial Medicine, of Illinois State Medical Society, for 1858—A Correction, etc."

I do not think it advisable to fill a medical journal with matters of personal controversy, but think a few words are called for in the present instance.

The reference to Dr. Hutchinson is found in the contribution of Dr. Payne, a member of the committee; and, however the mistake was made, the committee stand corrected. Allusion is made by Dr. Green, to what he considers an important omission in the report, viz.: the use of chlorate potassa in stomatitis materna. He is "wholly unprepared to assign reasons that influenced the committee to *ignore* a remedy which, of late, has received so much prominence in the hands of the profession."

I would call attention to what is made the duty, by the society, of the Committee on Practical Medicine: "They shall prepare an annual report on the most important improvements effected in *this State*, in the management of individual diseases, and on the progress of epidemics; referring, as occasion requires, to medical topography, and to the character of prevailing diseases in special localities, during the term of their service." Soon after their appointment, the committee issued circulars to the profession of the State, and caused a copy to be published in the Chicago Medical Journal. It will be seen, then, what would naturally constitute the material from which the report of the committee would be made.

The chairman, during the year, endeavored to glean from sources within the State, all that could be obtained, upon the subjects embraced in the circular. Chlorate of potassa received a share of notice, and all that could be collected from sources within the State, was presented. If Dr. Green, or any other member of the profession in Indiana, had found the medicine in question a valuable remedy in stomatitis materna, it was his duty to report it, and not lay a charge of discourtesy to a committee, a year after their report was written, for omitting what had not come to their notice.

Thinking the above remarks will show how far the committee were guilty of any want of regard to the profession, I remain

Very respectfully, Yours,

F. K. BAILEY,

Chairman of Committee, etc., for 1858.

JOLIET, April, 1859.

---

HANCOCK MEDICAL ASSOCIATION.

The seventh annual meeting of Hancock Medical Association was held in Warsaw, Wednesday, April 20th, 1859. Dr. Hollowbush in the chair. Dr. S. L. Comer was elected a member. The following were elected officers for the ensuing year:

| | |
|---|---|
| *President*, . . | Dr. H. Judd, Warsaw. |
| *Vice-President*, . . | A. Spitler, Carthage. |
| *Treasurer*, . . . | Chas. Hay, Warsaw. |
| *Secretary*, . . | Geo. W. Hall, Carthage. |

Delegates to the American Medical Association—David Ellis, M.D., W. M. King, M.D., and Geo. W. Hall, M.D.

Delegates to State Medical Society—H. Judd, M.D., and S. L. Comer, M.D.

On motion, the Secretary was directed to prepare an account of the meeting of the Association, for publication in the Chicago Medical Journal.

The Association adjourned to meet in Carthage, the third Wednesday in July. GEO. W. HALL, Secretary.

LITHOTOMY.

This operation was performed by Dr. Brainard, on the 10th of May, on a boy three years old, who is at the present time quite recovered. This is the sixteenth operation of Dr. Brainard, and as yet no accident has occurred in any one of them. This small number of cases indicates the great infrequency of urinary calculus in this region, as compared with other States, and particularly Kentucky. The operation preferred is the lateral, or if the stone be very large, the bi-lateral—the neck of the bladder being distended with the bistoury. P.

We see it stated, in the Press and Tribune of this city, that the new rooms for the medical department of the Lind University, are being put in order. They are to be in the upper story of Mr. Lind's warehouse, on the south branch of the Chicago River.

CORRECTION.—The Boston Medical and Surgical Journal, in a notice of our article on chronic hydrocephalus, speaks of Dr. Tournesko's case as the first. This is an error. Our own case preceded that of Dr. Tournesko five years.

Prof. John H. Rauch has resigned the chair of Materia Medica, in Rush Medical College.

EXCISED KNEES.

A most curious and novel sight occurred the other night, at the London Medical Society, which took my fancy very much. Mr. P. C. Price, a young surgeon of great promise, a protege of my friend Mr. Ferguson, and who has at the same time performed most of the capital operations, read a paper on some of the causes of failure following the operation of excision of the knee joint, in which he most ably considered the subject, and very satisfactorily showed that the want of a successful issue depended upon circumstances which might have occurred had amputation been performed. At the conclusion of his paper, some ten or more male and female persons walked into the room, each of whom had undergone resection of one of their knees, and who were living proofs of the value of a limb without a joint. One lad could walk his 14 miles a day, without inconvenience or fatigue—all were in excellent health. The sight was rather amusing too, for both males and females had their knees exposed whilst walking up and down to show their anarthrodial agility. Mr. Price is engaged in the preparation of a treatise on excision of the knee, which will be copiously illustrated, and at the same time will contain an account of every operation that has been done, up to the year 1858.—*Montreal Chronicle.*

